# Efficacy and Safety of Lenvatinib Combined With PD-1 Inhibitors Plus TACE for Unresectable Hepatocellular Carcinoma Patients in China Real-World

**DOI:** 10.3389/fonc.2022.950266

**Published:** 2022-07-04

**Authors:** Xiaowei Li, Zhigang Fu, Xiaoxia Chen, Kunkun Cao, Jiaming Zhong, Li Liu, Ning Ding, Xiaoli Zhang, Jian Zhai, Zengqiang Qu

**Affiliations:** Department II of Interventional Radiology, Eastern Hepatobiliary Surgery Hospital, Shanghai, China

**Keywords:** hepatocellular carcinoma, lenvatinib, immune checkpoint inhibitors, TACE, tumor number, neutrophil lymphocyte ratio

## Abstract

**Purpose:**

To evaluate the efficacy and safety of lenvatinib combined with programmed death receptor-1 signaling inhibitors plus transarterial chemoembolization (LePD1-TACE) for treatment of unresectable hepatocellular carcinoma (uHCC) in a real-world setting in China.

**Methods:**

This was a retrospective study involving consecutive patients with uHCC (n =114) receiving LePD1-TACE treatment from June 2019 to May 2021. Overall survival (OS), progression-free survival (PFS), objective response rate (ORR), and disease control rate (DCR) were calculated to evaluate the antitumor efficacy. Treatment-related adverse events (TRAEs) were analyzed to assess the safety profiles. In addition, we also evaluated prognostic factors related to survival and disease progression.

**Results:**

A total of 114 patients with a median age of 53 years were analyzed during a median follow-up duration of 10.6 months (95% confidence interval [CI]: 8.5 -12.8). The Kaplan-Meier analysis showed that the median OS was 18.0 months (95% CI: 14.1 - Not reached), the median PFS was 10.4 months (95% CI: 6.6 - 12.4). Based on modified Response Evaluation Criteria in Solid Tumors, the best ORR was 69.3% and DCR was 80.7%. Almost all patients suffered from TRAEs, the most common grade 3-4 TRAEs were hypertension (8.8%), proteinuria (3.6%), hyperbilirubinemia (1.8%), leukopenia (4.4%) and alanine aminotransferase elevation (3.6%) across all patients. The independent treatment factors associated with OS and PFS were tumor number, neutrophil-to-lymphocyte ratio (NLR) and the early tumor response. In the early tumor response (CR+PR) patients, median OS and PFS were 25.1 months (95% CI: 13.8 - Not reached) and 15.2 months (95% CI: 10.5 - 19.1). The patients with tumor number < 3 had a superior median OS and PFS (25.1, 16.4 months) compared to patients with tumor number ≥ 3 (14.1 months, P = 0.012; 6.6 months, P = 0.007). The patients with NLR ≤ 2.165 had a longer median OS and PFS (Not reached, 15.2 months) than those with NLR > 2.165 (17.7 months, P = 0.003; 7.5 months, P = 0.047).

**Conclusion:**

In this real-world study, LePD1-TACE triple therapy showed encouraging efficiency and manageable safety in patients with uHCC. The tumor number (< 3), NLR (≤ 2.165) and early tumor response (CR+PR) could be one of the prognostic markers.

## Introduction

Hepatocellular carcinoma (HCC) is the sixth most commonly malignancy and the third leading cause of cancer death (8.3% of the total cancer deaths) world-wide in 2020 (1). According to the Barcelona Clinic Liver Cancer (BCLC) staging system, early-stage HCC patients with good liver function should be treated with radical therapy, but patients with unresectable hepatocellular carcinoma (uHCC) are usually treated with chemoembolization and systemic therapies (2–4). Two randomized controlled trials have confirmed the effectiveness of transarterial chemoembolization (TACE) in the treatment of intermediate-stage HCC (5, 6), and in the real world, TACE is also commonly used for uHCC in Asia including China, Japan and Korea (7–10). Nowadays, six Food and Drug Administration approved systemic therapies including tyrosine kinase inhibitors (TKIs) and immune checkpoint inhibitors (ICIs) also have shown the promising approaches to uHCC (2, 11–17).

Lenvatinib, as a novel oral multi-kinase inhibitor, was demonstrated to be effective and well tolerated in a global open label randomized phase III study (REFLECT) (18). The final results established a significantly improved progression-free survival (PFS) for lenvatinib (7.4 months) compared with sorafenib (3.7 months), the median survival time was non-inferior to that with sorafenib (13.6 months versus 12.3 months). More recently, several ICIs are used in clinical trials, especially the programmed cell death protein-1 (PD-1) inhibitors, which block PD-1 or PD-L1 have proven a promising efficacy and safety in treatment of advanced HCC (11, 19). At least six native and non-native PD-1 inhibitors, including camrelizumab, sintilimab, toripalimab, tislelizumab, nivolumab and pembrolizumab are available for HCC patients in China (7, 20–22).

In clinical practice, as the number of systemic drugs and combination therapies continues to grow, the challenge is to determine which combination and sequence of treatments provides the best outcome with minimal toxicity. There are increasing clinical trials designed to investigate combination treatment regimens, including combining TKIs and ICIs, locoregional treatment and systemic therapies (23), but to date, few data have been published regarding outcomes achieved with lenvatinib-based triple combination therapy. The triple combination of lenvatinib, PD-1 inhibitors and TACE (LePD1-TACE) may have an enhanced clinical benefit. Therefore, we conducted this retrospective real-world data study to evaluate the efficacy and safety of LePD1-TACE for the treatment of uHCC in China. In addition, we also explored the prognostic factors related to the efficacy of LePD1-TACE treatment.

## Patients and Methods

### Study Design and Patients

**Figure 1:**
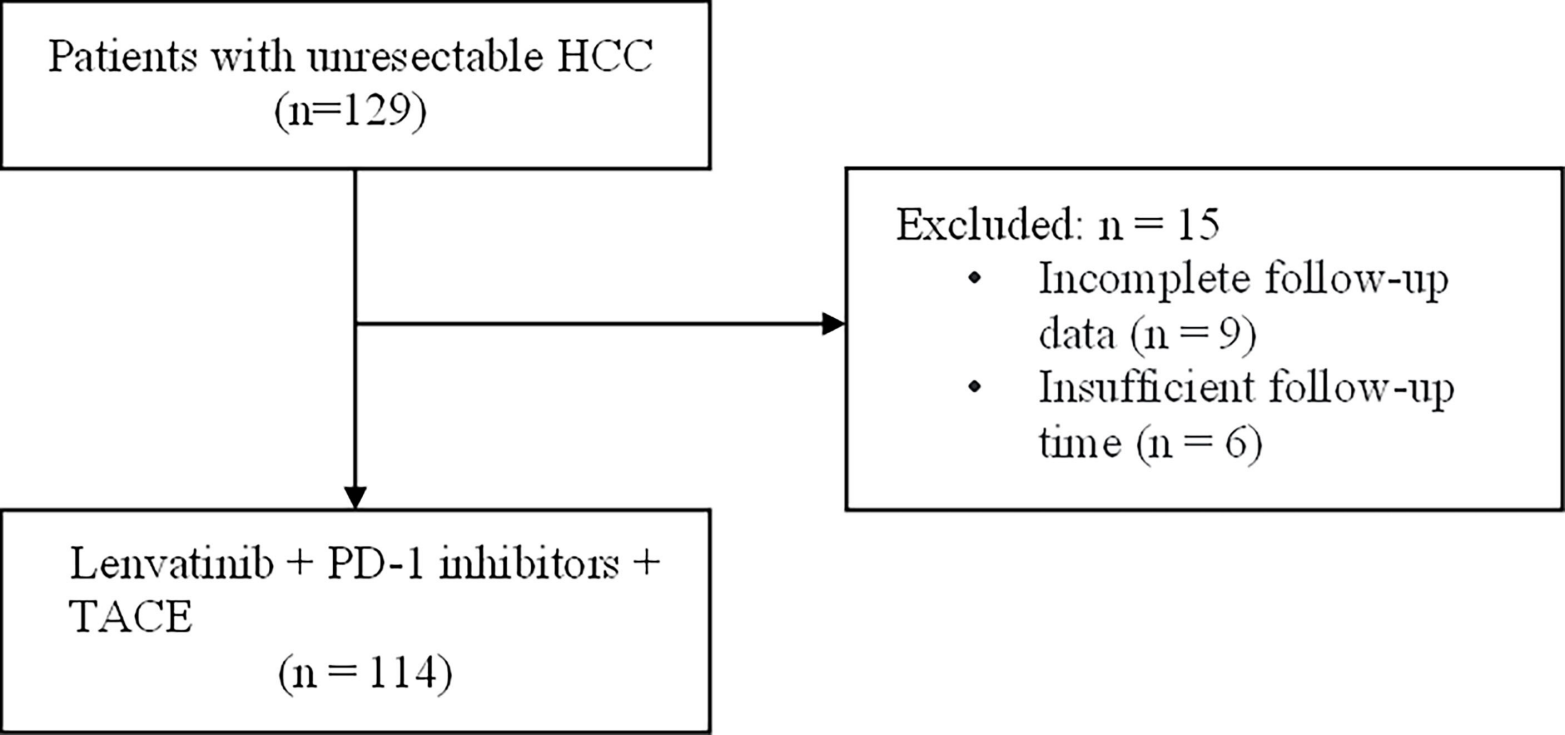
Flowchart of the patient selection process. HCC, hepatocellular carcinoma; PD-1 inhibitors, programmed death receptor-1 signaling inhibitors; TACE, transarterial chemoembolization.

The study was designed as an observational, hospital-based, retrospective cohort study. The medical records of consecutive uHCC (n = 129) patients who received LePD1-TACE treatment from June 2019 to May 2021. Diagnosis of HCC was based on pathological or non-invasive assessment according to criteria of 2019 HCC Guidelines in China (7), and the American Association for the Study of Liver Disease (24). Owing to the setting of the retrospective real-world study, only patients with incomplete follow-up data and insufficient follow-up time were excluded (n = 15) (**Figure 1**). This study was approved by the ethical committee of the Eastern Hepatobiliary Surgery Hospital and was performed in accordance with the Declaration of Helsinki. Written informed consent for the LePD1-TACE protocol was obtained from all enrolled patients.

### Treatment Protocol

For patients diagnosed with uHCC, lenvatinib was used as the first-line recommended therapy based on the current international guidelines. In addition, PD-1 inhibitors and TACE were also recommended. As we have previously demonstrated in a published study that lenvatinib and TACE combination therapy may significantly improve clinical outcomes compared to TACE monotherapy for uHCC with a manageable safety (25), so the lenvatinib-based triple combination treatment of LePD1-TACE might have a clinically meaningful improvement in antitumor activity. According to the principle of intent-to-treat, before treatment, patients were informed of any potential side effects and the cost of long-term treatment. The treatment protocols were reviewed by the attending physician and the final decision was principally made by the patient.

### LePD1-TACE Combination Therapy

For patients who received LePD1-TACE combination therapy, on-demand TACE was performed by a team of experienced physicians. The TACE procedure comprised intraarterial angiography to identify the main supplying artery of the tumor, followed by transarterial injection of a mixture of chemotherapy drug (pirarubicin) and lipiodol. Depending on tumor stain, injection of embolic gelatin sponges or blank microspheres to interrupt blood flow. Follow-up imaging was performed every 6-8 weeks after TACE treatment. TACE was repeated if it produced a tumor response of partial response (PR) or stable disease (SD), meanwhile the liver function was good and there was no evidence of hepatic decompensation. Lenvatinib and PD-1 inhibitors were administered concurrently 3-5 days after the first TACE treatment, based on the restored liver function after TACE and the patient’s general status. Patients received oral lenvatinib 8 mg (if body weight < 60 kg) or 12 mg (if body weight ≥ 60 kg) once daily and PD-1 inhibitors (camrelizumab 200 mg, toripalimab 240 mg, sintilimab 200 mg or tislelizumab 200 mg) intravenously every 3 weeks. To avoid additive adverse event (AE), lenvatinib was discontinued 2 days before and after each TACE procedure, and the PD-1 inhibitors should be delayed until 3 days after TACE if the timing of injection coincides with TACE. Dose reductions (to 8 mg and 4 mg/day, or 4 mg every other day) for lenvatinib-related AEs were allowed. For serious immune-related adverse events (irAEs), the PD-1 inhibitors were discontinued and the corticosteroids should be considered for treatment of irAEs.

### Evaluation of Treatment Response and Adverse Events

Tumor evaluation was performed at baseline and every 6-8 weeks using liver enhanced magnetic resonance imaging or computed tomography, alpha-fetoprotein (AFP), desgamma-carboxy prothrombin (DCP), and physical findings. Overall survival (OS) was defined as the time from initiation of LePD1-TACE triple combined treatment to death due to any cause. PFS was defined as the time from the initiation of the triple combined treatment to disease progression or death. Tumor response were based on the modified Response Evaluation Criteria in Solid Tumors 1.1 (mRECIST) (26) and immune-related RECIST (22). Patients were initially assessed for progression; imaging assessments were performed 4 weeks later to exclude possible pseudo-progression due to the PD-1 inhibitors in the combination treatment. The treatment response was defined as the best overall response throughout the follow-up period. Early response was defined as the radiological response at the first follow-up visit. Early AFP (baseline ≥ 25ng/mL) and DCP (baseline ≥ 40mAU/mL) responses were assessed at the first follow-up after LePD1-TACE induction, a positive response was defined as a reduction of ≥ 20% from baseline. The objective response rate (ORR) and the disease control rate (DCR) were defined as the proportion of patients with complete response (CR) or PR and CR, PR or SD, respectively. The incidence and severity of AEs complied with the criteria of the Common Terminology Criteria for Adverse Events (CTCAE, version 5.0). Mild post-TACE AEs such as nausea, fever, abdominal pain, and transient elevated liver enzymes including alanine aminotransferase (ALT)/aspartate aminotransferase (AST) were not included.

### Factors Associated With Overall Survival and Disease Progression

We evaluated the prognostic factors associated with survival and disease progression using the variables of sex, age, BCLC stage (B vs. C), tumor number, tumor size, up-to-seven criteria (in vs. out), macrovascular invasion, extrahepatic metastasis, baseline AFP, NLR and PLR. We also included early tumor response indicators such as early tumor response (CR + PR), early AFP response, and early DCP response as factors potentially associated with survival and progression. Finally, subgroup analysis was performed to determine whether potential prognostic factors influenced treatment outcomes.

### Statistical Analysis

All statistical analysis was performed using IBM SPSS software (ver. 25.0 SPSS Inc., Chicago, IL, USA). The continuous variables were presented as the median with interquartile range and categorical variables as counts with percentages. Survival analysis was conducted using the Kaplan-Meier method and a log-rank test was chosen to compare patients’ survival between subgroups. The Cox proportional hazards regression method was used to identify the factors associated with survival and disease progression, and multivariate analysis used the variables with p < 0.10 in the univariate analysis. p < 0.05 was considered significant.

## Results

### Patient Characteristics

The baseline characteristics of the enrolled patients are summarized in **Table 1**. The median age was 53 years (range 24 – 79 years), and 102 of the patients (89.5%) were male, with a male to female ratio of 8.5:1. Most patients 89.5% (102/114) were positive for hepatitis B surface antigen (HBsAg), and 1.8% (2/114) for hepatitis C antibody. Sixty-nine (60.5%) patients were diagnosed with BCLC stage C and 42 (36.8%) with BCLC stage B, while 3 (2.6%) patients were BCLC stage A. The majority of patients were classified as Child-Pugh A (n = 111, 97.4%), and 3 patients (2.6%) had Child-Pugh B. Tumor number of 1,2, and ≥ 3 were reported for 17.5%, 18.4%, and 64.1% of patients, with the median tumor size was 82mm (range 13 – 216mm). The proportions of patients with macrovascular invasion and extrahepatic metastasis were observed at 48.3% and 20.2%, respectively. The median AFP level was 386 ng/mL (interquartile range [IQR], 20.5 – 7130.5), and DCP level was 2833 mAU/mL (IQR, 385 – 13805). The laboratory indicators at baseline including total bilirubin (TB), albumin (ALB), ALT, white blood cell (WBC), platelet (PLT) counts, and neutrophil lymphocyte ratio (NLR), platelet lymphocyte ratio (PLR) were also summarized (**Table 1**).

**Table 1:** Baseline characteristics of the 114 patients.

Characteristics	Statistical value
Median age, years (range)	53 (24–79)
Male gender	102 (89.5)
Etiology	
Hepatitis B	102 (89.5)
Hepatitis C	2 (1.8)
Non-B, Non-C	10 (8.8)
BCLC stage	
A	3 (2.6)
B	42 (36.8)
C	69 (60.5)
Child-Pugh class	
A	111 (97.4)
B	3 (2.6)
Tumor number	
1	20 (17.5)
2	21(18.4)
≥3	73 (64.1)
Maximum tumor size, mm (range)	82 (13-216)
Macrovascular invasion	
Presence	55 (48.3)
Absence	59 (51.7)
Extrahepatic Metastases	
Presence	23 (20.2)
Absence	91 (79.8)
AFP (ng/mL), median (Q1, Q3)	386 (20.5, 7130.5)
≥25	81 (71.1)
DCP (mAU/mL), median (Q1, Q3)	2833 (385, 13805)
≥40	100 (87.7)
TB (μmol/L), median (Q1, Q3)	15 (11, 20)
ALB (g/L), median (Q1, Q3)	40 (37, 42)
ALT (U/L), median (Q1, Q3)	37 (27, 53)
WBC (×109),median (Q1, Q3)	5.26 (4.22, 6.59)
PLT (×109),median (Q1, Q3)	143 (112, 213)
NLR, median (Q1, Q3)	2.61 (1.94, 3.32)
PLR, median (Q1, Q3)	125.66 (91.80, 163.37)
**Prior treatment before LePD1-TACE**	
TACE	12 (10.5)
TACE-MWA	5 (4.4)
TACE-lenvatinib/sorafenib	7 (6.1)
TACE-MWA-lenvatinib	2 (1.8)
TACE-radiotherapy/lenvatinib	6 (5.3)
**Treatment after LePD1-TACE**	
Lenvatinib - PD-1	5 (4.4)
Regorafenib -TACE - PD-1	7 (6.1)
TACE- atezolizumab- bevacizumab	2 (1.8)
Radiotherapy- lenvatinib - PD-1	4 (3.5)
Others	7 (6.1)

Values are presented as median (range) or n (%). BCLC, Barcelona Clinic Liver Cancer; AFP, alpha-fetoprotein; DCP, Des-gammacarboxy; TB, total bilirubin; ALB, albumin; ALT, alanine aminotransferase; WBC, white blood cell; PLT, platelet; NLR, neutrophil lymphocyte ratio; PLR, platelet lymphocyte ratio; TACE, transarterial chemoembolization; MWA, microwave ablation; PD-1, programmed death receptor-1 signaling inhibitors.

Thirty-two (28.1%) patients had experienced prior treatment including TACE (n = 12), TACE-microwave ablation (MWA) (n = 5), TACE-lenvatinib (n = 4), TACE-MWA-lenvatinib (n = 2), TACE-radiotherapy (n = 3), TACE-sorafenib (n = 3), and TACE-radiotherapy-lenvatinib (n = 3). The median duration of lenvatinib was 8.13 months (IQR 6.5–11.9), and the mean interval between each TACE treatment was 101.3 days. Four types of PD-1 inhibitors were used in the combination therapy, including camrelizumab (n = 75, 65.7%), sintilimab (n = 22, 19.3%), toripalimab (n = 4, 3.6%), and tislelizumab (n = 13, 11.4%).

### Treatment Response and Survival Outcomes

**Figure 2:**
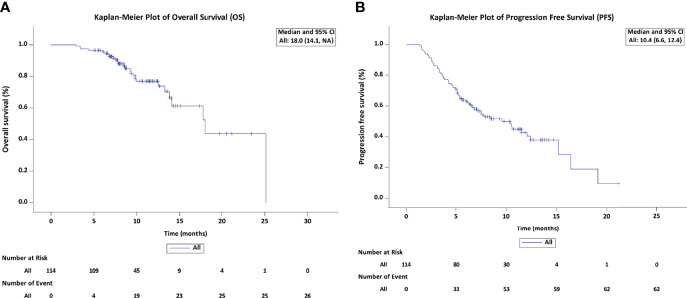
Kaplan-Meier estimates of overall survival **(A)** and progression-free survival **(B)** in patients treated with LePD1-TACE. LePD1-TACE, lenvatinib and programmed death receptor-1 signaling inhibitors plus transarterial chemoembolization.

**Figure 3:**
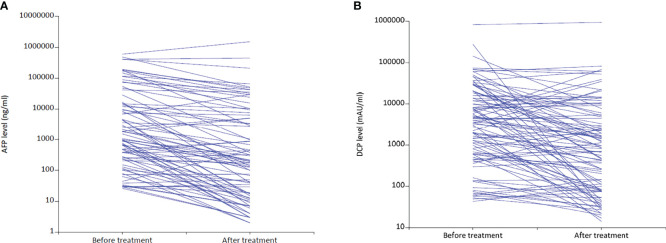
Changes in AFP **(A)** and DCP **(B)** levels from baseline to the first follow-up after LePD1-TACE treatment. AFP, alpha-fetoprotein; DCP, desgamma-carboxy prothrombin. LePD1-TACE, lenvatinib and programmed death receptor-1 signaling inhibitors plus transarterial chemoembolization.

By the censoring date of November 9, 2021, the median follow-up duration was 10.6 months (95% confidence interval [CI]: 8.5 – 12.8). The median PFS was 10.4 months (95% CI: 6.6 – 12.4), and the median OS was 18.0 months (95% CI: 14.1– Not reached) (**Figure 2**). The best tumor response was shown in **Table 2**. Based on the mRECIST criteria, 9 (7.9%) patients achieved CR, 70 (61.4%) patients achieved PR, 13 (11.4%) patients were SD, and 22 (19.3%) patients were PD. The best ORR and DCR were 69.3% (n = 79) and 80.7% (n = 92), respectively. We also analyzed the early changes in tumor response and marker from baseline to the first clinical evaluation. The early tumor response (CR+PR vs. SD+PD) was 65.8% (n = 75) vs. 34.2% (n = 39). Early positive AFP and DCP responses were 76.2% (64/84) and 62.7% (64/102), respectively (**Figure 3**).

**Table 2:** Best tumor response of the 114 patients.

Variable	Total (n = 114)
Best overall response by mRECIST version 1.1, n (%)	
Complete response	9 (7.9)
Partial response	70 (61.4)
Stable disease	13 (11.4)
Progressive disease	22 (19.3)
Objective response rate, n (%)	79 (69.3)
Disease control rate, n (%)	92 (80.7)

mRECIST, modified Response Evaluation Criteria in Solid Tumors 1.1.

There were 27 (23.7%) patients discontinued LePD1-TACE treatment mainly because of disease progression or serious TRAEs. At the end of the last follow-up, radiological disease progression was evident in 50 patients, 25 of whom continued to receive LePD1-TACE treatment and 25 did not. Subsequent treatments received by the 18 patients included lenvatinib - PD-1(n = 5), regorafenib -TACE - PD-1(n = 7), TACE plus atezolizumab and bevacizumab therapy (n = 2), radiotherapy- lenvatinib - PD-1(n = 4) **Table 1**.

### Safety Assessment

No treatment-related deaths occurred in this study, and treatment-related adverse events (TRAEs) were assessed mainly according to frequency and severity grade based on CTCAE, version 5.0. Most patients might develop mild post-TACE AEs, such as nausea, fever, abdominal pain, and transient elevated ALT and AST, which did not require any special treatment. Most patients improved within 1 week. Therefore, we did not include these mild AEs in this study.

As shown in **Table 3**, the most frequent TRAEs of all grades in LePD1-TACE treatment were hypertension (27.2%), hand-foot skin reaction (14.9%), diarrhea (12.3%), skin rash (13.2%), proteinuria (12.3%). The most frequent laboratory-related AEs of all grades were decreased albumin (42.1%), WBC (37.7%) and PLT (36.8%), increased ALT (15.8%), TB (12.3%), myocardial enzymes (12.3%) and thyroid-stimulating hormone (34.2%). In addition, the most frequent grade 3/4 AEs were hypertension (8.8%), proteinuria (3.6%), hyperbilirubinemia (1.8%), leukopenia (4.4%). Most of TRAEs could be managed by dose reduction, interruption, or standard medical treatment. Dose reduction or interruption was observed in 21 (18.4%) of patients during the study period. None of the patients discontinued lenvatinib because of AEs, however, there were 2 (1.8%) patients discontinued PD-1 inhibitors owing to grade 3 immune-related AEs of hepatitis.

**Table 3:** Treatment-related adverse events.

Adverse events	All grades, n (%)		Grade 3/4, n (%)
Hypertension	31 (27.2)		10 (8.8)
Hand-foot skin reaction	17 (14.9)		2 (1.8)
Diarrhea	14 (12.3)		1 (0.9)
Skin rash	15 (13.2)		2 (1.8)
Proteinuria	14 (12.3)		4 (3.6)
RCCEP	5 (4.4)		0 (0)
Fatigue	9 (7.9)		1 (0.9)
Epistaxis	1 (0.9)		0 (0)
Joint pain	6 (5.3)		0 (0)
Bleeding (gingiva)	11 (9.6)		2 (1.8)
Dysphonia	4 (3.6)		0 (0)
Anorexia	6 (5.3)		0 (0)
Myocardial enzymes elevation	14 (12.3)		0 (0)
Hypothyroidism	39 (34.2)		0 (0)
AST elevation	16 (14)		4 (3.6)
ALT elevation	18 (15.8)		2 (1.8)
Hyperbilirubinemia	14 (12.3)		2 (1.8)
Decreased albumin	48 (42.1)		0 (0)
Decreased PLT	42 (36.8)		2 (1.8)
Decreased WBC	43 (37.7)		5 (4.4)
Interruption and/or dose reduction		21 (18.4)	
Discontinued PD-1 inhibitors		2 (1.8)	

RCCEP, reactive cutaneous capillary endothelial proliferation; AST, aspartate aminotransferase; ALT, alanine aminotransferase, PLT, platelet; WBC white blood cell; PD-1 inhibitors, programmed death receptor-1 signaling inhibitors.

At baseline, all patients were tested for hepatitis markers and viral load, and those with antiviral indications received antiviral therapy before treatment. Patients who had hepatitis B virus (HBV) infection received tenofovir (TDF) or entecavir (ETV) treatment, and sofosbuvir for hepatitis C virus infection. Considering the ICIs, antiviral regimens such as interferon that might affect immune modulation were not used by patients. Increased HBV-DNA concentrations were detected in 2 (1.8%) patients during follow-up, patients had their therapeutic regimen changed (ETV switched to TDF). No patients interrupted or discontinued LePD1-TACE treatment owing to HBV-DNA increase.

### Factors Associated With Overall Survival and Disease Progression

The independent factors predictive of survival and disease progression based on univariate and multivariate Cox regression analyses are summarized in **Table 4** and **Table 5**. In univariable analysis, tumor number, up-to-seven criteria (in vs. out), NLR, PLR and the early tumor response (CR+PR vs. SD+PD) were significantly associated with OS. In multivariable analysis, pre-treatment factors of the tumor number ≥ 3 (vs. < 3; hazard ratio [HR] = 0.22, 95% CI: 0.08-0.66; p = 0.0069), NLR ≤ 2.165(vs. > 2.165; HR = 14.91, 95% CI: 1.99-111.79; p = 0.0086) and post-treatment factor of the early tumor response CR+PR (vs. SD+PD; HR = 2.64, 95% CI: 1.04-6.75; p = 0.0419) were independent factors predictive of OS. In univariable analysis, tumor number, NLR, early AFP and DCP response and early tumor response (CR+PR vs. SD+PD) were significantly associated with PFS. In multivariable analysis, the tumor number ≥ 3 (vs. < 3; HR = 0.39, 95% CI: 0.21-0.71; p = 0.0021), NLR ≤ 2.165(vs. > 2.165; HR = 2.34, 95% CI: 1.25-4.37; p = 0.0078), and the early tumor response CR+PR (vs. SD+PD; HR = 7.56, 95% CI: 3.81-14.98; p < 0.0001) were independent factors predictive of PFS.

**Table 4:** Multivariable Cox regression analysis for overall survival.

Variables	Univariate Analysis		Multivariate Analysis	
	HR (95% CI)	P value	HR (95% CI)	P value
Gender (Male vs. female)	1.22 (0.42 -3.58)	0.7147		
Age (≤53 vs. >53)	0.77 (0.34-1.75)	0.5264		
BCLC stage (B vs. C)	1.79 (0.73-4.34)	0.1950		
Tumor number (≥3 vs.< 3)	**0.27 (0.09-0.81)**	**0.0120**	**0.22 (0.08- 0.66)**	**0.0069**
Largest tumor size (<82 vs. ≥82)	1.61 (0.71-3.63)	0.2497		
Up-to-seven criteria (in vs. out)	**3.53 (1.03-12.07)**	**0.0331**		
Baseline AFP (<400 vs. ≥400)	1.78 (0.79- 4.00)	0.1579		
NLR (≤ 2.165 vs. >2.165)	**11.29 (1.52-83.82)**	**0.0028**	**14.91 (1.99-111.79)**	**0.0086**
PLR (<96.42 vs. ≥96.42)	**3.33 (0.99-11.19)**	**0.0393**		
Extrahepatic metastasis (absent vs. presence)	1.74 (0.75-4.05)	0.1913		
Macrovascular invasion (absent vs. present)	1.55 (0.69-3.47)	0.2851		
Early AFP response (decrease ≤20% vs. >20%)	1.23 (0.44-3.45)	0.6919		
Early DCP response (decrease ≤20% vs. >20%)	0.98 (0.41-2.36)	0.9727		
Early tumor response (CR+PR vs. SD+PD)	**2.61 (1.18-5.74)**	**0.0137**	**2.64 (1.04-6.75)**	**0.0419**

HR, hazard ratio; CI, confidence interval; BCLC, Barcelona Clinic Liver Cancer; AFP, alpha-fetoprotein; DCP, Des-gammacarboxy; NLR, neutrophil lymphocyte ratio; PLR, platelet lymphocyte ratio; CR, complete response; PR, partial response; SD, stable disease; PD, progressive disease.

**Table 5:** Multivariable Cox regression analysis for progression-free survival.

Variables	Univariate Analysis		Multivariate Analysis	
	HR (95% CI)	P value	HR (95% CI)	P value
Gender (Male vs. female)	1.14 (0.54-2.42)	0.7231		
Age (≤53 vs. >53)	0.66 (0.40-1.10)	0.1071		
BCLC stage (B vs. C)	1.05 (0.63-1.77)	0.8412		
Tumor number (≥3 vs.< 3)	**0.47 (0.27-0.82)**	**0.0066**	**0.39 (0.21-0.71)**	**0.0021**
Largest tumor size (<82 vs. ≥82)	1.14 (0.69-1.88)	0.6152		
Up-to-seven criteria (in vs. out)	0.98 (0.56-1.72)	0.9437		
Baseline AFP (<400 vs. ≥400)	1.43 (0.86-2.40)	0.1664		
NLR (≤2.165 vs. >2.165)	**1.82 (1.00-3.33)**	**0.0466**	**2.34 (1.25-4.37)**	**0.0078**
PLR (<96.42 vs. ≥96.42)	1.30 (0.73-2.32)	0.3658		
Extrahepatic metastasis (absent vs. presence)	1.56 (0.89-2.74)	0.1182		
Macrovascular invasion (absent vs. present)	0.94 (0.57-1.56)	0.8137		
Early AFP response (decrease ≤20% vs. >20%)	**0.39 (0.21- 0.72)**	**0.0020**		
Early DCP response (decrease ≤20% vs. >20%)	**0.43 (0.25- 0.74)**	**0.0015**		
Early tumor response (CR+PR vs. SD+PD)	**5.45 (3.22- 9.21)**	**<.0001**	**7.56 (3.81- 14.98)**	**<.0001**

HR, hazard ratio; CI, confidence interval; BCLC, Barcelona Clinic Liver Cancer; AFP, alpha-fetoprotein; DCP, Des-gammacarboxy; NLR, neutrophil lymphocyte ratio; PLR, platelet lymphocyte ratio; CR, complete response; PR, partial response; SD, stable disease; PD, progressive disease.

**Figure 4:**
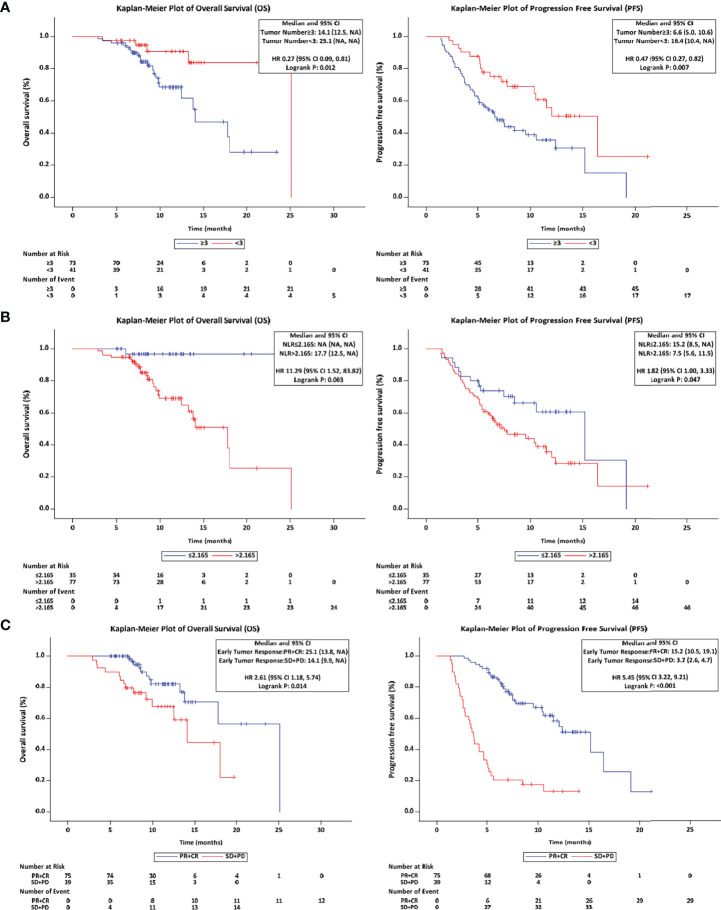
Overall and progression-free survival with different stratified. **(A)** The OS and PFS in tumor number (≥ 3 vs. < 3) patients. **(B)** The OS and PFS in NLR (≤ 2.165 vs. > 2.165) patients. **(C)** The OS and PFS in early tumor response (CR+PR vs. SD+PD) patients. OS, overall survival; PFS, progression-free survival; NLR, neutrophil lymphocyte ratio; CR, complete response; PR, partial response; SD, stable disease; PD, progressive disease.

Considering the tumor number, NLR and early tumor response were independent predictors of OS and PFS after LePD1-TACE treatment, we further analyzed the treatment outcomes stratified by tumor number (≥ 3 vs. < 3), NLR (≤ 2.165 vs. > 2.165) and early tumor response (CR+PR vs. SD+PD). The patients with tumor number < 3 had a superior median OS and PFS (25.1, 16.4 months) compared to patients with tumor number ≥ 3 (14.1 months, P = 0.012; 6.6 months, P = 0.007; **Figure 4A**). The patients with NLR ≤ 2.165 had a longer median OS and PFS (Not reached, 15.2 months) than those with NLR > 2.165 (17.7 months, P = 0.003; 7.5 months, P = 0.047; **Figure 4B**). The patients with early tumor response (CR+PR) also exhibited a longer median OS and PFS (25.1, 15.2 months) compared with their counterparts (14.1 months, p = 0.014; 3.7 months, p < 0.0001; **Figure 4C**).

## Discussion

Several retrospective real‐world studies from Korea and Japan supported the efficacy of lenvatinib in patients who exceeded the REFLECT trial criteria (e.g., ≥ 50% liver involvement, main portal vein invasion and extrahepatic metastasis) (27–29). However, in clinical practice, uHCC is typically characterized by high tumor burdens, high invasiveness, rapid progress and poor prognosis. Most patients with uHCC receive a combination of therapies, including lenvatinib, during their lifespan, which may or may not have a survival benefit. Randomized controlled studies of the combination therapy based on lenvatinib are underway (NCT04246177, NCT04997850, NCT04974281, NCT04273100), but data on LePD1-TACE triple therapy are still scarce. Therefore, we conducted this real-world study on the efficacy and safety of LePD1-TACE in Chinese uHCC patients.

In our present study, LePD1-TACE triple therapy showed a favorable efficacy and manageable toxicity in patients with uHCC in a real-world setting. The results showed a high ORR and DCR according to mRECIST of 69.3% and 80.7%, median PFS of 10.4 months, and median OS was 18.0 months. As we reported previously, lenvatinib-TACE contributed to longer OS and PFS than TACE monotherapy, and ORR was better at 68.3% (25). Triple therapy did not appear to be significantly superior to double in ORR. Given the nature of real-world study, all patients using the triple combination regimen between June, 2019 and May, 2021 were included in this study without strict screening and exclusion criteria, therefore, it could better reflect the actual clinical therapeutic effect.

In fact, the patients enrolled in this study had worse tumor biological behaviors with higher proportions of macrovascular invasion (48.3% vs. 21%) and extrahepatic metastases (20.2% vs. 15%) compared to the patients enrolled in previous study (25). This might be the cause of the result. In addition, compared with other studies in the literature, the PFS of lenvatinib-TACE was 5.5 -11.6 months (30–32), while the PFS of our LePD1-TACE triple treatment achieved 10.4 months, our results were in the upper range. Notably, the different results between these studies and our data should be viewed with caution.

Recently, lenvatinib in combination with the PD-1 inhibitor pembrolizumab showed encouraging antitumor activity in a phase I studies, the ORR (RECIST 1.1) was 36% (33). Further validation was underway in the Phase III randomized clinical trial (LEAP-002; NCT03713593). The immunomodulatory effects of lenvatinib on tumor microenvironment might contribute to PD-1 inhibitor antitumor activity (34). In the TACTICS trial (35), sorafenib (a multikinase inhibitor) pretreatment 2 - 3 weeks before initial TACE to normalize the tumor vasculature, which significantly improved the primary endpoint of PFS in patients with uHCC. Based on the results of the above double combination and our triple combination study, it is believed that the combination of lenvatinib, PD-1 inhibitor and TACE may have a synergistic antitumor effect.

First, TACE induces a microenvironment of ischemia and hypoxia, leading to tumor necrosis and tumor-specific antigen release. The combination of PD1 inhibitor may enhance the development of tumor antigen specific memory T cells and maintain the anti-tumor response of patients (36). Hypoxic microenvironment promotes up-regulation of hypoxia inducible factor-1 alpha, bFGF, and VEGF, while combined with lenvatinib inhibits the activity of tumor angiogenesis factors (18, 37). Second, lenvatinib plus PD-1 inhibitor exerts unique immunomodulatory effects by blocking FGFR-4, reducing Treg differentiation and inhibiting TGFß signaling (38, 39). Lenvatinib also contributes to vascular normalization and promotes immune cell infiltration into the tumor (36, 40). In conclusion, the triple therapy regimen produces a positive immune profile and a faster therapeutic response.

This study also assessed the potential toxicity or side effects of combination therapy. Despite the combination of lenvatinib and PD-1 inhibitors, which have different pharmacological mechanisms, no new safety-related events were identified in this study. Most AEs were mild to moderate and easily manageable with appropriate supportive care. A total of 367 AEs occurred at all grades, and 37 AEs were observed at grade 3-4. The albumin and WBC decreased were the most common AEs, occurred in 42.1%, 37.7% of patients, respectively, and hypertension (8.8%), proteinuria (3.6%), and WBC decreased (4.4%) were the most frequent grade 3-4 AEs. We also observed a decrease in TSH (34.2%), which was higher than in the REFLECT trial and might be related to the combination of PD-1 inhibitors. In addition, increased HBV-DNA was observed in only 2 patients during treatment, indicating that the combination therapy did not significantly activate the hepatitis B virus, possibly because of the use of potent antiviral drugs.

Of note, two patients underwent conversion resection after PR. Both patients were BCLC A, and their tumors shrank significantly after treatment and met the criteria for surgical resection. Our results indicated that the conversion rates are not high in the real-world. Although resection criteria were met, some patients refused surgical resection and continued triple therapy. The efficacy of conversion resection remains controversial, but the high ORR of triple therapy (69.3%) might be a potential option for conversion therapy. Nine patients who achieved CR were followed regularly and maintained dual therapy with Lenvatinib and PD-1 inhibitors.

In the present study, the tumor number < 3, NLR ≤ 2.165 and early tumor response (CR+PR) were identified as the independent predictors of OS and PFS. As an indicator of tumor-related inflammation, NLR is able to predict tumor prognosis. A high NLR is associated with increased systemic inflammation and reduced cancer-specific immunity (downregulation of tumor-infiltrating lymphocytes, for instance) (41). In patients who undergo TACE for intermediate-stage HCC, higher NLRs are associated with poor prognosis (42). In several studies, the NLR cutoff value for HCC ranges from 2 to 5 (42–44). In this study, patients with baseline NLR ≤ 2.165 before LePD1-TACE had longer PFS and OS than patients with NLR > 2.165. Subgroup analyses also showed that the patients with tumor number < 3 had a superior median OS and PFS compared to patients with tumor number ≥ 3. Due to the limitations of this single-arm study, the effect of LePD1-TACE on patients with tumor number ≥ 3, NLR > 2.165, and early tumor response (PD+SD) needs to be further studied, LePD1-TACE may provide a greater survival benefit in these populations than advanced -stage standard therapy. A previous retrospective study reported that LePD1-TACE significantly improved survival over lenvatinib-TACE in advanced HCC patients with tumor number > 3 but without main portal vein invasion (45). It is useful to predict effective cases at an early stage when considering the complexity of liver cancer and possible side effects. Early tumor response (CR+PR) suggested that the treatment was effective, and provided evidence for the next treatment strategy for patients.

There are several limitations worthy of mention. Firstly, in view of the retrospective, single-hospital study, there may be inherent information and selection bias, especially when comparing groups, unknown differences between the two groups may bias the results. Secondly, our study was a single-arm design and was limited by the lack of a control group. Zhu et al. (31) reported that the effectiveness of triple therapy (Lenvatinib + PD-1 inhibitors + TACE or HAIC) is not superior to dual therapy (Lenvatinib + PD-1 inhibitors or Lenvatinib + TACE or HAIC). Therefore, multicenter randomized controlled clinical trials need to be carried out to verify the efficacy and safety of different combinations. Thirdly, a variety of PD-1 inhibitors were used in this study, which affected the consistency of treatment regiments.

In conclusion, our real-world study showed that LePD1-TACE triple therapy was well tolerated with encouraging efficacy in patients with uHCC. The patients with the tumor number (< 3), NLR ≤ 2.165 and early tumor response (CR+PR) might achieve better clinical outcomes.

## Data Availability Statement

The raw data supporting the conclusions of this article will be made available by the authors, without undue reservation.

## Ethics Statement

This study was approved by the ethical committee of the Eastern Hepatobiliary Surgery Hospital and was performed in accordance with the Declaration of Helsinki. Written informed consent was obtained from all enrolled patients.

## Author Contributions

XL, ZF, JZ and ZQ conceived and designed research. XL, MZ, XC, KC, ND, LL, XZ collected and assembled the data. XL, JZ and ZQ performed or supervised analyses. MZ, XC interpreted the results. KC, LL, XZ, ZF performed statistical expertise. XL, MZ, ND, ZF wrote sections of the initial draft. JZ and ZQ provided substantive suggestions for revision. JZ, KC, LL, ND, XZ, MZ, XC provide the provision of study materials or patients. All reviewed and approved final version of the paper

## Conflict of Interest

The authors declare that the research was conducted in the absence of any commercial or financial relationships that could be construed as a potential conflict of interest.

## Publisher’s Note

All claims expressed in this article are solely those of the authors and do not necessarily represent those of their affiliated organizations, or those of the publisher, the editors and the reviewers. Any product that may be evaluated in this article, or claim that may be made by its manufacturer, is not guaranteed or endorsed by the publisher.

## References

[1] Sung H Ferlay J Siegel RL Laversanne M Soerjomataram I Jemal A. Global Cancer Statistics 2020: GLOBOCAN Estimates of Incidence and Mortality Worldwide for 36 Cancers in 185 Countries. CA Cancer J Clin (2021) 71(3):209–49. doi: 10.3322/caac.21660 33538338

[2] Llovet JM Kelley RK Villanueva A Singal AG Pikarsky E Roayaie S. Hepatocellular Carcinoma. Nat Rev Dis Primers (2021) 7(1):6. doi: 10.1038/s41572-020-00240-3 33479224 PMC13537433

[3] European Association for the Study of the Liver. EASL Clinical Practice Guidelines: Management of hepatocellular carcinoma. J Hepatol (2018) 69 (1):182–236. doi: 10.1016/j.jhep.2018.03.019 29628281

[4] Vogel A Martinelli E clinicalguidelines@esmo.org EGCEa, Committee EG. Updated Treatment Recommendations for Hepatocellular Carcinoma (HCC) From the ESMO Clinical Practice Guidelines. Ann Oncol (2021) 32(6):801–5. doi: 10.1016/j.annonc.2021.02.014 33716105

[5] Llovet JM Real MI Montaña X Planas R Coll S Aponte J. Arterial Embolisation or Chemoembolisation Versus Symptomatic Treatment in Patients With Unresectable Hepatocellular Carcinoma: A Randomised Controlled Trial. Lancet (2002) 359(9319):1734–9. doi: 10.1016/S0140-6736(02)08649-X 12049862

[6] Lo CM Ngan H Tso WK Liu CL Lam CM Poon RT. Randomized Controlled Trial of Transarterial Lipiodol Chemoembolization for Unresectable Hepatocellular Carcinoma. Hepatology (2002) 35(5):1164–71. doi: 10.1053/jhep.2002.33156 11981766

[7] Zhou J Sun H Wang Z Cong W Wang J Zeng M. Guidelines for the Diagnosis and Treatment of Hepatocellular Carcinoma (2019 Edition). Liver Cancer (2020) 9(6):682–720. doi: 10.1159/000509424 33442540 PMC7768108

[8] Kudo M Kawamura Y Hasegawa K Tateishi R Kariyama K Shiina S. Management of Hepatocellular Carcinoma in Japan: JSH Consensus Statements and Recommendations 2021 Update. Liver Cancer (2021) 10(3):181–223. doi: 10.1159/000514174 34239808 PMC8237791

[9] Korean Liver Cancer Association National Cancer Center. 2018 Korean Liver Cancer Association-National Cancer Center Korea Practice Guidelines for the Management of Hepatocellular Carcinoma. Gut Liver (2019) 13(3):227–99. doi: 10.5009/gnl19024 PMC6529163 31060120

[10] Feng F Jiang Q Jia H Sun H Chai Y Li X. Which is the Best Combination of TACE and Sorafenib for Advanced Hepatocellular Carcinoma Treatment? A Systematic Review and Network Meta-Analysis. Pharmacol Res (2018) 135:89–101. doi: 10.1016/j.phrs.2018.06.021 29959032

[11] El-Khoueiry AB Sangro B Yau T Crocenzi TS Kudo M Hsu C. Nivolumab in Patients With Advanced Hepatocellular Carcinoma (CheckMate 040): An Open-Label, non-Comparative, Phase 1/2 Dose Escalation and Expansion Trial. Lancet (2017) 389(10088):2492–502. doi: 10.1016/S0140-6736(17)31046-2 PMC7539326 28434648

[12] Vogel A Qin S Kudo M Su Y Hudgens S Yamashita T. Lenvatinib Versus Sorafenib for First-Line Treatment of Unresectable Hepatocellular Carcinoma: Patient-Reported Outcomes From a Randomised, Open-Label, non-Inferiority, Phase 3 Trial. Lancet Gastroenterol Hepatol (2021) 6(8):649–58. doi: 10.1016/S2468-1253(21)00110-2 34087115

[13] Zhu AX Kang Y-K Yen C-J Finn RS Galle PR Llovet JM. Ramucirumab After Sorafenib in Patients With Advanced Hepatocellular Carcinoma and Increased α-Fetoprotein Concentrations (REACH-2): A Randomised, Double-Blind, Placebo-Controlled, Phase 3 Trial. Lancet Oncol (2019) 20(2):282–96. doi: 10.1016/S1470-2045(18)30937-9 30665869

[14] Finn RS Qin S Ikeda M Galle PR Ducreux M Kim TY. Atezolizumab Plus Bevacizumab in Unresectable Hepatocellular Carcinoma. N Engl J Med (2020) 382(20):1894–905. doi: 10.1056/NEJMoa1915745 32402160

[15] Llovet JM Ricci S Mazzaferro V Hilgard P Gane E Blanc JF. Sorafenib in Advanced Hepatocellular Carcinoma. N Engl J Med (2008) 359(4):378–90. doi: 10.1056/NEJMoa0708857 18650514

[16] Bruix J Qin S Merle P Granito A Huang Y-H Bodoky G. Regorafenib for Patients With Hepatocellular Carcinoma Who Progressed on Sorafenib Treatment (RESORCE): A Randomised, Double-Blind, Placebo-Controlled, Phase 3 Trial. Lancet (2017) 389(10064):56–66. doi: 10.1016/S0140-6736(16)32453-9 27932229

[17] Roskoski R Jr. Properties of FDA-Approved Small Molecule Protein Kinase Inhibitors: A 2022 Update. Pharmacol Res (2022) 175:106037. doi: 10.1016/j.phrs.2021.106037 34921994

[18] Kudo M Finn RS Qin S Han K-H Ikeda K Piscaglia F. Lenvatinib Versus Sorafenib in First-Line Treatment of Patients With Unresectable Hepatocellular Carcinoma: A Randomised Phase 3 non-Inferiority Trial. Lancet (2018) 391(10126):1163–73. doi: 10.1016/S0140-6736(18)30207-1 29433850

[19] Zhu AX Finn RS Edeline J Cattan S Ogasawara S Palmer D. Pembrolizumab in Patients With Advanced Hepatocellular Carcinoma Previously Treated With Sorafenib (KEYNOTE-224): A non-Randomised, Open-Label Phase 2 Trial. Lancet Oncol (2018) 19(7):940–52. doi: 10.1016/S1470-2045(18)30351-6 29875066

[20] Xie DY Ren ZG Zhou J Fan J Gao Q. 2019 Chinese Clinical Guidelines for the Management of Hepatocellular Carcinoma: Updates and Insights. Hepatobiliary Surg Nutr (2020) 9(4):452–63. doi: 10.21037/hbsn-20-480 PMC7423548 32832496

[21] Qin S Ren Z Meng Z Chen Z Chai X Xiong J. Camrelizumab in Patients With Previously Treated Advanced Hepatocellular Carcinoma: A Multicentre, Open-Label, Parallel-Group, Randomised, Phase 2 Trial. Lancet Oncol (2020) 21(4):571–80. doi: 10.1016/S1470-2045(20)30011-5 32112738

[22] Ren Z Xu J Bai Y Xu A Cang S Du C. Sintilimab Plus a Bevacizumab Biosimilar (IBI305) Versus Sorafenib in Unresectable Hepatocellular Carcinoma (ORIENT-32): A Randomised, Open-Label, Phase 2–3 Study. Lancet Oncol (2021) 22(7):977–90. doi: 10.1016/S1470-2045(21)00252-7 34143971

[23] Hui F Xu C Xu X Chen J Geng H Yang C. What Is the Most Suitable Agent Combined With Apatinib for Transarterial Chemoembolization Treatment in Advanced Hepatocellular Carcinoma Patients? A Systematic Review and Network Meta-Analysis. Front Oncol (2022) 12. doi: 10.3389/fonc.2022.887332 PMC9174538 35692745

[24] Marrero JA Kulik LM Sirlin CB Zhu AX Finn RS Abecassis MM. Diagnosis, Staging, and Management of Hepatocellular Carcinoma: 2018 Practice Guidance by the American Association for the Study of Liver Diseases. Hepatology (2018) 68(2):723–50. doi: 10.1002/hep.29913 29624699

[25] Fu Z Li X Zhong J Chen X Cao K Ding N. Lenvatinib in Combination With Transarterial Chemoembolization for Treatment of Unresectable Hepatocellular Carcinoma (uHCC): A Retrospective Controlled Study. Hepatol Int (2021) 15(3):663–75. doi: 10.1007/s12072-021-10184-9 PMC8286947 33877527

[26] Llovet JM Lencioni R. mRECIST for HCC: Performance and Novel Refinements. J Hepatol (2020) 72(2):288–306. doi: 10.1016/j.jhep.2019.09.026 31954493 PMC12452114

[27] Tsuchiya K Kurosaki M Sakamoto A Marusawa H Kojima Y Hasebe C. The Real-World Data in Japanese Patients With Unresectable Hepatocellular Carcinoma Treated With Lenvatinib From a Nationwide Multicenter Study. Cancers (Basel) (2021) 13(11):2608. doi: 10.3390/cancers13112608 34073396 PMC8198233

[28] Sho T Suda G Ogawa K Shigesawa T Suzuki K Nakamura A. Lenvatinib in Patients With Unresectable Hepatocellular Carcinoma Who do Not Meet the REFLECT Trial Eligibility Criteria. Hepatol Res (2020) 50(8):966–77. doi: 10.1111/hepr.13511 32562334

[29] Cheon J Chon HJ Bang Y Park NH Shin JW Kim KM. Real-World Efficacy and Safety of Lenvatinib in Korean Patients With Advanced Hepatocellular Carcinoma: A Multicenter Retrospective Analysis. Liver Cancer (2020) 9(5):613–24. doi: 10.1159/000508901 PMC7548882 33083284

[30] Chen S Wu Z Shi F Mai Q Wang L Wang F. Lenvatinib Plus TACE With or Without Pembrolizumab for the Treatment of Initially Unresectable Hepatocellular Carcinoma Harbouring PD-L1 Expression: A Retrospective Study. J Cancer Res Clin Oncol (2021). doi: 10.1007/s00432-021-03767-4 PMC9293824 34453221

[31] Zhu Y Sun P Wang K Xiao S Cheng Y Li X. Efficacy and Safety of Lenvatinib Monotreatment and Lenvatinib-Based Combination Therapy for Patients With Unresectable Hepatocellular Carcinoma: A Retrospective, Real-World Study in China. Cancer Cell Int (2021) 21(1):503. doi: 10.1186/s12935-021-02200-7 34537075 PMC8449874

[32] Ando Y Kawaoka T Amioka K Naruto K Ogawa Y Yoshikawa Y. Efficacy and Safety of Lenvatinib-Transcatheter Arterial Chemoembolization Sequential Therapy for Patients With Intermediate-Stage Hepatocellular Carcinoma. Oncology (2021) 99(8):507–17. doi: 10.1159/000515865 33946070

[33] Finn RS Ikeda M Zhu AX Sung MW Baron AD Kudo M. Phase Ib Study of Lenvatinib Plus Pembrolizumab in Patients With Unresectable Hepatocellular Carcinoma. J Clin Oncol (2020) 38(26):2960–70. doi: 10.1200/JCO.20.00808 PMC7479760 32716739

[34] Faivre S Rimassa L Finn RS. Molecular Therapies for HCC: Looking Outside the Box. J Hepatol (2020) 72(2):342–52. doi: 10.1016/j.jhep.2019.09.010 31954496

[35] Kudo M Ueshima K Ikeda M Torimura T Tanabe N Aikata H. Randomised, Multicentre Prospective Trial of Transarterial Chemoembolisation (TACE) Plus Sorafenib as Compared With TACE Alone in Patients With Hepatocellular Carcinoma: TACTICS Trial. Gut (2020) 69(8):1492–501. doi: 10.1136/gutjnl-2019-318934 PMC7398460 31801872

[36] Cheu JW Wong CC. Mechanistic Rationales Guiding Combination Hepatocellular Carcinoma Therapies Involving Immune Checkpoint Inhibitors. Hepatology (2021) 74(4):2264–76. doi: 10.1002/hep.31840 33811765

[37] Wu JY Yin ZY Bai YN Chen YF Zhou SQ Wang SJ. Lenvatinib Combined With Anti-PD-1 Antibodies Plus Transcatheter Arterial Chemoembolization for Unresectable Hepatocellular Carcinoma: A Multicenter Retrospective Study. J Hepatocell Carcinoma (2021) 8:1233–40. doi: 10.2147/JHC.S332420 PMC8502053 34676181

[38] Yi C Chen L Lin Z Liu L Shao W Zhang R. Lenvatinib Targets FGF Receptor 4 to Enhance Antitumor Immune Response of Anti-Programmed Cell Death-1 in HCC. Hepatology (2021) 74(5):2544–60. doi: 10.1002/hep.31921 34036623

[39] Torrens L Montironi C Puigvehi M Mesropian A Leslie J Haber PK. Immunomodulatory Effects of Lenvatinib Plus Anti-Programmed Cell Death Protein 1 in Mice and Rationale for Patient Enrichment in Hepatocellular Carcinoma. Hepatology (2021) 74(5):2652–69. doi: 10.1002/hep.32023 PMC12155127 34157147

[40] Lu Y Jin J Du Q Hu M Wei Y Wang M. Multi-Omics Analysis of the Anti-Tumor Synergistic Mechanism and Potential Application of Immune Checkpoint Blockade Combined With Lenvatinib. Front. Cell Dev. Biol. (2021) 9:730240. doi: 10.3389/fcell.2021.730240 34568339 PMC8458708

[41] Coussens LM Werb Z. Inflammation and Cancer. Nature (2002) 420(6917):860–7. doi: 10.1038/nature01322 PMC2803035 12490959

[42] Chu HH Kim JH Shim JH Gwon DI Ko HK Shin JH. Neutrophil-To-Lymphocyte Ratio as a Biomarker Predicting Overall Survival After Chemoembolization for Intermediate-Stage Hepatocellular Carcinoma. Cancers (Basel) (2021) 13(11):2830. doi: 10.3390/cancers13112830 34204125 PMC8201147

[43] Limaye AR Clark V Soldevila-Pico C Morelli G Suman A Firpi R. Neutrophil-Lymphocyte Ratio Predicts Overall and Recurrence-Free Survival After Liver Transplantation for Hepatocellular Carcinoma. Hepatol Res (2013) 43(7):757–64. doi: 10.1111/hepr.12019 PMC3622781 23193965

[44] Zhou D Zhang Y Xu L Zhou Z Huang J Chen M. A Monocyte/Granulocyte to Lymphocyte Ratio Predicts Survival in Patients With Hepatocellular Carcinoma. Sci Rep (2015) 5:15263. doi: 10.1038/srep15263 26486016 PMC4614102

[45] Cai M Huang W Huang J Shi W Guo Y Liang L. Transarterial Chemoembolization Combined With Lenvatinib Plus PD-1 Inhibitor for Advanced Hepatocellular Carcinoma: A Retrospective Cohort Study. Front Immunol (2022) 13:848387. doi: 10.3389/fimmu.2022.848387 35300325 PMC8921060

